# Digital disconnection as a self-regulatory strategy against procrastination

**DOI:** 10.1038/s41598-026-46218-1

**Published:** 2026-05-29

**Authors:** Julius Klingelhoefer, Alicia Gilbert, Adrian Meier

**Affiliations:** 1 School of Business, Economics and Society, Friedrich-Alexander-Universität Erlangen- Nürnberg, Findelgasse 7-9, 90402 Nürnberg, Germany; 2 Department of Communication, Johannes Gutenberg-Universität Mainz, Mainz, Germany

**Keywords:** digital disconnection, procrastination, self-regulation, goal conflict, self-control, Neuroscience, Psychology, Psychology

## Abstract

Digital media are pervasive and offer various short-term rewards, yet they may undermine the achievement of long-term goals by fostering *procrastination*—the irrational delay of intended tasks. Research documents that media users try to avoid procrastination through *digital disconnection*, the voluntary and temporary reduction of digital media use. Combining theoretical perspectives on psychological processes of self-regulation and digital disconnection, our study investigates whether goal conflicts and trait self-control make engaging in disconnection more likely and whether disconnection is in turn associated with less procrastination. In an experience sampling study with *N* = 237 young adult participants and *T* = 12,408 observations, we find that in situations in which participants experienced stronger goal conflicts they were more likely to engage in digital disconnection and that individuals who generally experience higher goal conflict were more likely to disconnect. Additionally, those with higher trait self-control disconnected more. Most notably, disconnection was associated with lower procrastination on the within-person level: In situations with more disconnection, procrastination was lower. Exploratory analyses find that this effect persisted to the following situation. Our results show that engaging in digital disconnection could be a promising self-control strategy to reduce procrastination.

Whether at work or in their free time, many report the same experience: the moment they intend to get started on a task, a notification, feed, or screen pulls their attention elsewhere. This “attention economy” is characterized by an environment of constant connectivity^1^, with enjoyable, entertaining, and engaging activities always just a swipe away. Part of a broader “techlash”, users are therefore increasingly frustrated with digital media and try out the occasional “digital detox”. Indeed, ubiquitous connectivity can and does regularly challenge individuals’ capabilities to successfully complete personally important tasks, particularly in work and learning environments^2–4^, but also in other spheres of life including health and social relations^5,6^. Specifically, many perceive issues with *procrastination*, i.e., the delay of intended tasks, which entails prioritizing short-term rewards over long-term goals despite expecting to be worse off overall^7^.

While procrastination is prevalent in many life domains^5^, the context of digital media use can serve as an informative case in highly digitalized societies. Generally, studies find that more frequent use of smartphones, social media, or games is associated with more procrastination^8,9^. This can be explained by the formation of powerful usage habits, driven by persuasive technology design^10,11^. Stronger habits, such as regularly checking one’s phone or social media, are in turn consistently associated with more procrastination among adolescents^12^, college students^13^, and working adults^14^.

Accordingly, people often limit their usage of digital technologies, trying to not “waste time,” reduce interruptions, or be less distracted^15–17^. Such behaviors are defined as *digital disconnection*, that is, deliberate actions aimed at temporarily abstaining from or reducing digital media use. Whether disconnection can be an effective self-control strategy to reduce procrastination, however, remains largely untested. In this paper, we take a perspective that focuses on researching how individual psychological functioning can be improved^18^. While researchers criticize that discourse around self-regulation of digital technologies often reproduces individual responsibilization of systemic issues^3^, we argue that it is also important to improve individuals’ strategies to deal with negative aspects they face with digital media. Here, we offer a comprehensive investigation of digital disconnection as a self-control strategy against procrastination. While an early field experiment found that procrastination was reduced during a short break from social media or video games^19^, such “digital detox” intervention studies have been questioned for their validity^20,21^. Additionally, media users can apply multiple disconnection strategies^15^, which might differ in their effectiveness. Accordingly, we investigate “life as it is lived”^22^ by assessing whether engaging in various disconnection strategies in daily life is associated with reduced procrastination. We conduct a two-week experience sampling study with *N* = 237 young adult participants and *T* = 12,408 situational measurements.

## Digital disconnection and procrastination

Digital disconnection can be understood as an attempt to self-regulate digital media use^23^. *Self-regulation*, the process of monitoring and taking action towards achieving a desired goal^6^, is a relevant skill in many areas of life. Self-regulation plays an essential role when tasks are aversive and do not offer an immediate or high enough reward, such as doing chores, studying, or working^6^. *Self-control* as a specific form of self-regulation is activated when two *goals* compete for resources (e.g., time, energy) and a *goal conflict* arises as a result^6^. In the case of procrastination, individuals are typically confronted with the choice to engage in immediately gratifying activities (*desires*) or tasks that help achieve personally or socially important long-term goal*s*, rendering the conflicting desire a *temptation*^24^. Digital media, for example, can increase procrastination by affording constant and immediate access to various emotional, excitative, or social desires^2,8,25^, for example, when someone scrolls through social media feeds despite the need to call their relative or clean the house.

Procrastination means “to voluntarily delay an intended course of action despite expecting to be worse off for the delay”^7^. Thus, conflicts between short-term desires and long-term goals are particularly relevant to procrastination^6^. In such situations, procrastination occurs when individuals engage in a desired activity instead of pursuing a long-term goal that is viewed as important, resulting in a failed resolution of the goal conflict because the intention does not align with the action^25^. Thus, procrastination is often characterized as *irrational delay* and a form of *self-control failure*^7,26^. Here, we view irrationality from a perspective of instrumental rationality, that is whether goals are (mis-)aligned with behaviors and constitute an intention-action gap^27,28^. Which goals are perceived as desirable over others, and thus the appraisal of what constitutes a needless delay, depends on individual and societal contexts. Additionally, social norms, commonly understood as social expectations around behavior, can shape goal hierarchies. Consequently the perception of violating normative expectations by delaying or not achieving a goal can illicit negative emotional reactions^13,29^. While particularly in Western and highly digitalized societies, norms around procrastination are often framed around productivity, especially in the context of work and studying, procrastination can also happen in the context of health-related behaviors such as exercise, social relationships, or self-fulfillment^5,6,30^. Importantly, not all delay is procrastination; delaying a task on purpose, such as taking breaks for recovery or waiting for more information to complete the task, is usually understood as *rational delay* and not considered in this study^31^.

According to the *process model of self-control*, individuals engage in different strategies to avoid self-control failures such as procrastination^23,32^. *Situational strategies* can entail either preventing or modifying situations in which a desire conflicts with a goal^32^. In the context of digital disconnection this could mean that someone leaves their phone at home or turns it off to avoid the temptation of using it. *Intrapsychic strategies*, in turn, aim at modulating the response to conflicts between goals and temptation by deploying attention elsewhere, changing cognitions, or inhibiting one’s response^32^. For disconnection this could mean deliberately trying to avoid temptations by diverting attention away from a group chat or actively suppressing the habit of reaching towards the smartphone by taking a pen. Accordingly, disconnection can aim at preventing future unwanted media use by modifying upcoming situations or it can address an acute goal conflict with behavioral strategies that deliberately disengage from ongoing media use^33^.

Digital disconnection strategies employed for self-regulation can target different aspects of media use, with varying relevance for outcomes like well-being or productivity^17^. Based on the Hierarchical Computer Mediated Communication (CMC) Taxonomy^34^, digital disconnection can occur at different levels of media use^16,35^. Disconnection can refer to reductions in usage of entire (1) devices, e.g., smartphone, laptop, or smartwatch, (2) certain (types of) applications, e.g., all social media, only TikTok or YouTube, (3) individual features, e.g., turning off notifications or muting chats, (4) particular interactions, e.g., work or personal contacts, or (5) certain messages, e.g., immersive or entertaining content. Disconnection at each of these levels may be uniquely (in)effective in reducing procrastination. The recently proposed process-based framework of digital disconnection^17^, for instance, argues that disconnection strategies at higher levels of the taxonomy (i.e., device, application) may be more effective at shutting out many distractors at once, because all lower levels are nested in them. When putting one’s phone in another room, for example, a person does not need to block certain apps or mute notifications as the phone is out of reach.

Yet, evidence on the effectiveness of these various disconnection strategies, interventions, and behaviors against procrastination is limited. Two early studies by Hinsch and Sheldon^19^ showed that reducing Facebook use and social online gaming for two days was associated with less procrastination. However, a recent study by Klingelhoefer et al.^36^ found no evidence that employing more disconnection strategies was linked to lower distraction or higher productivity one month later. Because procrastination and task engagement considerably depend on situational context^8,32,37^, investigating the short-term, momentary associations of digital disconnection and procrastination seems pertinent. To our knowledge, this is the first naturalistic study that observes if voluntary digital disconnection is associated with reduced procrastination in daily life. Based on the process model of self-control, the multilevel approach to digital disconnection, and prior empirical studies, we argue that in situations in which participants deliberately reduce their usage of digital media, procrastination should be lower. Furthermore, procrastination should be lower for those who, on average, engage in more disconnection:

### H1

Digital disconnection is negatively associated with procrastination (a) within and (b) between participants.

However, likely not everybody employs disconnection strategies equally. On the trait level, self-control is an important predictor of beneficial outcomes like well-being or employment^38^. *Self-control* can be viewed as the individual capacity to resolve conflicts between competing goals in a preventive or interventive way^6^. This means that individuals with high trait self-control apply strategies, first, to arrange their goals and activities to encounter less goal conflicts in daily life and second, if conflicts do arise, enact strategies that resolve acute goal conflicts^39^. Because digital disconnection is frequently employed with the motivation to increase productivity^15^ and marketed by the “digital wellness” industry as a solution to curb distractions and improve well-being^3,40^, it can be viewed as one such strategy. Thus, we argue that individuals higher in self-control should be more likely to engage in digital disconnection:

### H2

Trait self-control is associated with a higher likelihood of digital disconnection.

Additionally, Vanden Abeele et al.^17^ argue that disconnection should be especially important when goal conflicts occur. Because individuals usually do not intend to neglect long-term goals, digital media-induced goal conflict can lead to undesirable outcomes, such as negative self-conscious emotions, delayed bedtime, or lower performance^41–43^. To avoid negative consequences of self-regulatory failure, individuals are more likely to employ self-control strategies when they perceive goal conflicts^2,24,44^. Indeed, experience sampling research has found that goal conflict makes it likelier that people exercise self-control^45^. Thus, we predict that both for individuals who experience more goal conflicts as well as for situations in which goal conflicts are stronger, disconnection will be more likely:

### H3

Goal conflict is associated with a higher likelihood of digital disconnection (a) within and (b) between participants.

Finally, trait self-control is likely to influence how someone reacts to goal conflict. In other words, whether someone will execute behavior that is consistent with achieving short-term vs. long-term goals should depend on the interplay between trait self-control and the occurrence of a goal conflict^24^. Indeed, studies find that in situations when long-term goals and short-term desires conflict, individuals with higher self-control capacity are more likely to engage in self-control strategies^46–48^. Therefore, engaging in digital disconnection as a self-control strategy should be more likely when high trait self-control coincides with a strong situational goal conflict.

### H4

There is a cross-level interaction effect of goal conflict and trait self-control on digital disconnection such that a combination of high goal conflict and high trait self-control is associated with a higher likelihood of digital disconnection.

## Method

To test our hypotheses, we conducted a two-week experience sampling method (ESM) study with up to five short surveys per day. The study was pre-registered here: https://osf.io/46rmg. We recruited young adults aged 18–35 using an academic online access panel (*SoSci Panel*)^49^ and e-mail lists at two German universities. We used the *movisensXS* experience sampling app, which only works on Android devices. In total, *N* = 237 Android smartphone users fulfilled our inclusion criteria (see preregistration) and provided *T* = 12,408 valid situational measurements. 156 participants identified as women, 77 as men, and four as nonbinary or another gender.

### Informed consent

about the procedure and content of the study was obtained from all participants prior to study participation. Our research was observational in nature and carried out in accordance with the guidelines of the School of Business, Economics and Society, Friedrich-Alexander-Universität Erlangen-Nürnberg and the guidelines by the DFG (German Research Foundation)^50^ which do not require ethical approval for studies that are non-experimental, do not involve vulnerable groups, physical or psychological risks, blinding, deception, or exceptional risks. The study was reviewed and approved by independent experts from the SoSci Panel^51^. When joining the study, participants filled out a pre-survey that included person-level measurements and received instructions for installing the app movisensXS. The app provider ensured compliance with data protection regulations (particularly GDPR) through technical and organizational measures. The app was used to send out short surveys (~ 1 min.) over the following 14 days at randomized time points from 9:00 to 21:00, with surveys at least two hours apart. To reduce interference with participants’ use or intentional non-use of digital media at the time of receiving the survey, our situational self-report measures focused on the last two hours.

In the ESM surveys, *procrastination* was measured with the item “In the last two hours, I needlessly delayed working on a task” (translated from German) adapted from Klein et al.^52^ and Sirois et al.^53^ on a scale from 1 (*not at all*) to 7 (*completely*). *Goal conflict* was measured with the question “In the last two hours, how much did your use of digital media conflict with other important goals such as work, studying, interactions, sports, etc.?” (translated from German) based on Halfmann et al.^41^ and Gilbert et al.^54^ on a scale from 1 (*not at all*) to 7 (*completely*). *Digital disconnection* was measured by assessing whether participants deliberately limited their digital media use at any of five levels of the CMC Taxonomy^34^, namely at the device, application, feature, interaction, and message content level. As pre-registered, for our confirmatory analyses, we created a binary variable of digital disconnection. If disconnection was reported at any level, it was scored at 1, if no disconnection occurred, it was scored as 0. Disconnection occurred in 7,360 situations (59%). For exploratory analyses, we also use a measure that reflects the breadth of disconnection behavior by averaging across all five disconnection levels (e.g., device, application, feature)^15^. Internal consistency for this measure was good (ω_within_ = 0.82, ω_between_ = 0.92). Trait *self-control* was measured with a German translation^55^ of the eight-item Brief-Self-Control Scale^56^. An example item is “I am good at resisting temptation”, which participants could rate from 1 (*completely disagree*) to 7 (*completely agree*). Internal consistency of this trait self-control index was high (ω_t_ = 0.88). Table 1 depicts descriptive statistics, correlations, and ICCs for central variables.

**Table 1:** *Descriptive statistics*,* within- and between-person correlations for central variables*.

Variable	Descriptives		Correlations^a^				ICC
	M	SD	1.	2.	3.	4.	
1. Self-control^b^	3.78	1.16	-	-	-	-	1
2. Goal conflict	3.19	2.13	− 0.22***	-	0.13***	0.31***	0.47
3. Disconnection^c^	-	-	0.14*	0.36***	-	− 0.09***	0.41
4. Procrastination	2.27	1.76	− 0.37***	0.56***	0.05	-	0.25

*Notes. N* = 237, *T* = 12,408. *p < .05, **p < .01, ***p < .001.

^a^ Within-person correlations depicted above, between-person correlations below the diagonal.

^b^ Self-control was measured as a trait, and no within-person correlation is available.

^c^ Digital disconnection was operationalized as a binary variable and does not have a mean or standard deviation.

## Results

For analyses, we used the R package *lme4*^57^ and constructed multilevel models that employ centering to separate variance of repeated measures into between- and within-person levels^58^. When predicting binary variables (i.e., digital disconnection), we utilized a logistic (i.e., nonlinear) function and report standardized effect sizes as odds ratios. For Likert-type measures, we utilize linear regression and report beta coefficients. As exploratory analyses, we investigated whether our predictors also significantly predict outcomes in the following situation. We additionally explored indirect effects utilizing multilevel structural equation modelling with the *lavaan* package^59^. Finally, we explored at which levels (e.g., device, feature) digital disconnection is associated with procrastination.

### Digital disconnection and procrastination

**Fig. 1:**
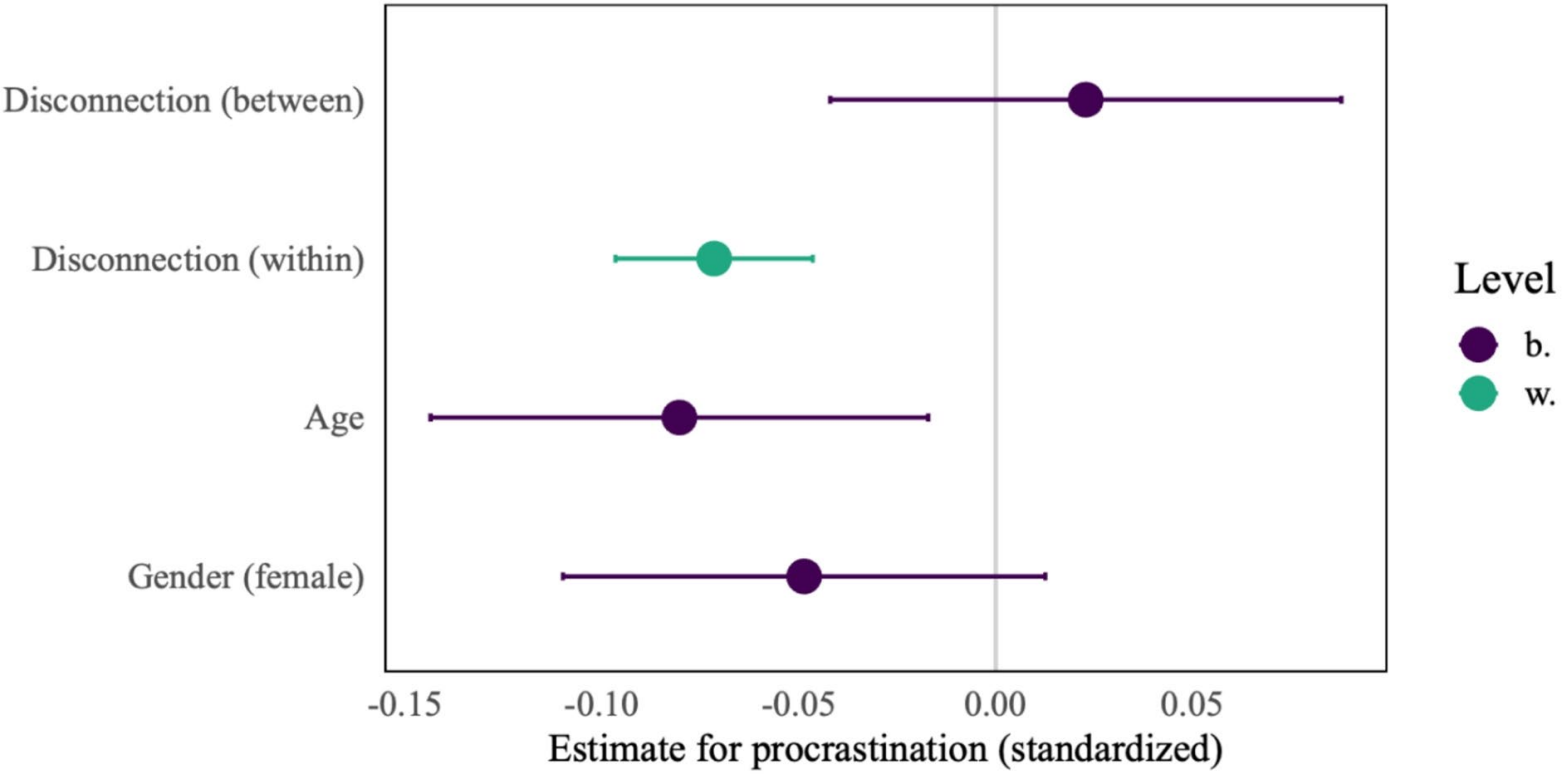
Multilevel model predicting procrastination with age and gender as control variables. *Notes*. 95% CI. b. = between, w. = within. *N* = 237, *T* = 12,408, ICC = 0.32, *R*^2^_marginal_ = 0.01, *R*^2^_conditional_ = 0.33.

To assess H1, a multilevel model with digital disconnection as predictor, procrastination as outcome, and age and gender as controls was constructed. The model is depicted in Fig. 1. Likelihood ratio tests show that a model with varying slopes is favored. On the within-person level, our model indicates that in situations in which participants engage in digital disconnection, they procrastinate slightly less, *b* = − 0.31, β = − 0.07, 95% CI [-0.10 – − 0.05], *p* < .001. Between participants, for those who were overall more likely to disconnect procrastination was not significantly lower, *b* = 0.13, β = 0.02, 95% CI [-0.04 – 0.09], *p* = .490. Thus, we find support for H1a (within-person) but not for H1b (between-person).

As a further exploration of this finding, we constructed a multilevel model that mirrors the model in Fig. 1 but predicts procrastination based on disconnection in the previous situation (*t*-1). Lending further support for H1a, the lag model indicates that digital disconnection in the previous situation significantly, yet weakly, predicted less procrastination over time (i.e., in the next measurement occasion), *b* = − 0.12, β = − 0.03, 95% CI [-0.04 – − 0.01], *p* = .011.

### Goal Conflict, self-control and digital disconnection

**Fig. 2:**
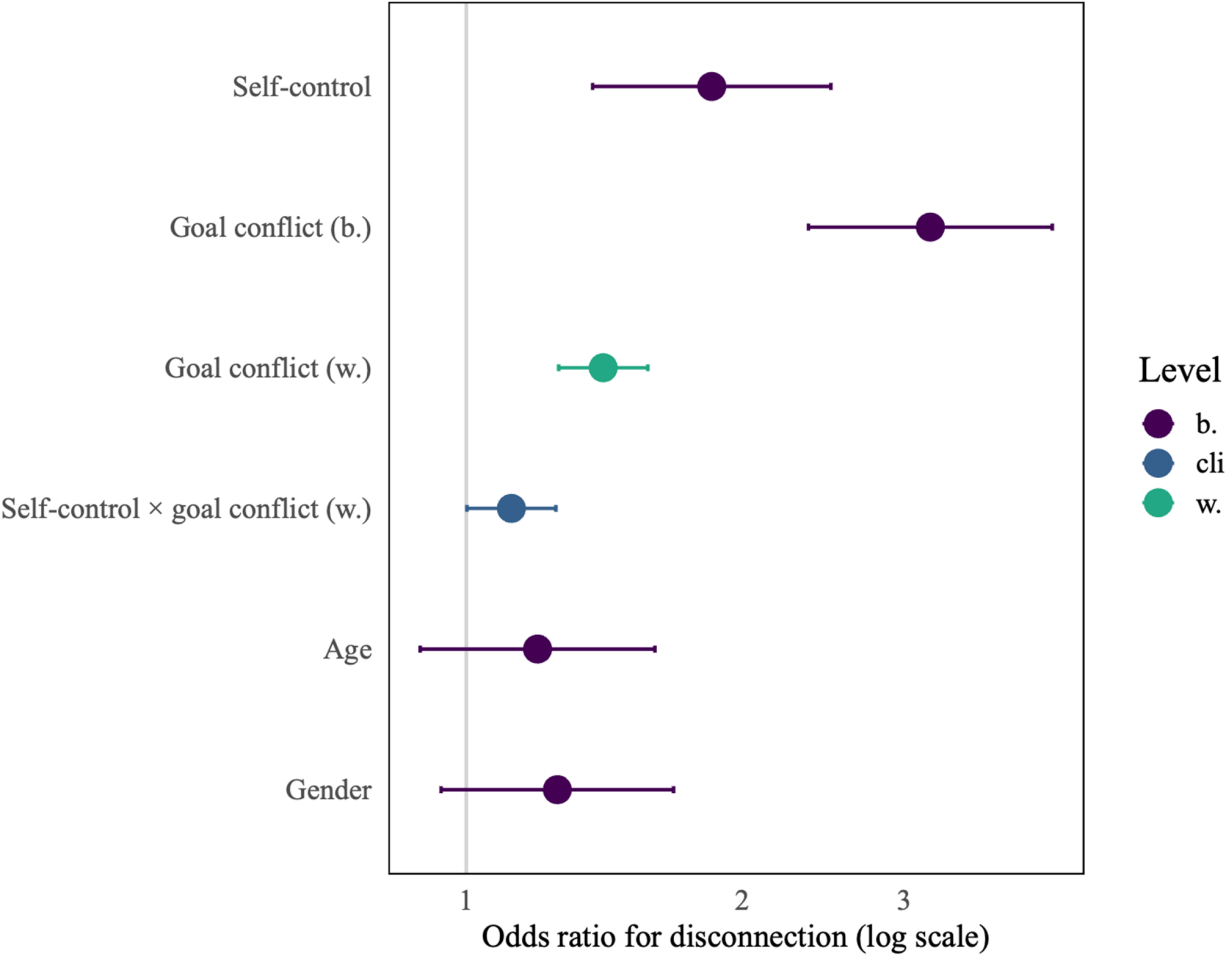
Multilevel model predicting digital disconnection with age and gender as control variables. *Notes*. 95% CI. b. = between, cli = within-between cross-level interaction, w. = within. *N* = 237, *T* = 12,408, ICC = 0.62, *R*^2^_marginal_ = 0.16, *R*^2^_conditional_ = 0.68.

To test whether digital disconnection is related to trait self-control (H2), goal conflict (H3), and the interaction between situational goal conflict and trait self-control (H4), we created a logistic multilevel model with digital disconnection as the binary outcome. The model is depicted in Fig. 2 and shows that those with one standard deviation higher trait self-control than others are 85% more likely to engage in disconnection behavior, *b* = 0.62, OR = 1.85, 95% CI [1.37–2.50], *p* < .001, supporting H2. In support for H3a, we find that in situations in which goal conflict is higher, disconnection is 41% more likely, *b* = 0.34, OR = 1.41, 95% CI [1.37–2.50], *p* < .001. Similarly, participants who on average experienced one standard deviation more goal conflict than others were more than thrice as likely to engage in digital disconnection (*b* = 1.16, OR = 3.20, 95% CI [2.36–4.35], *p* < .001), supporting H3b.

**Fig. 3:**
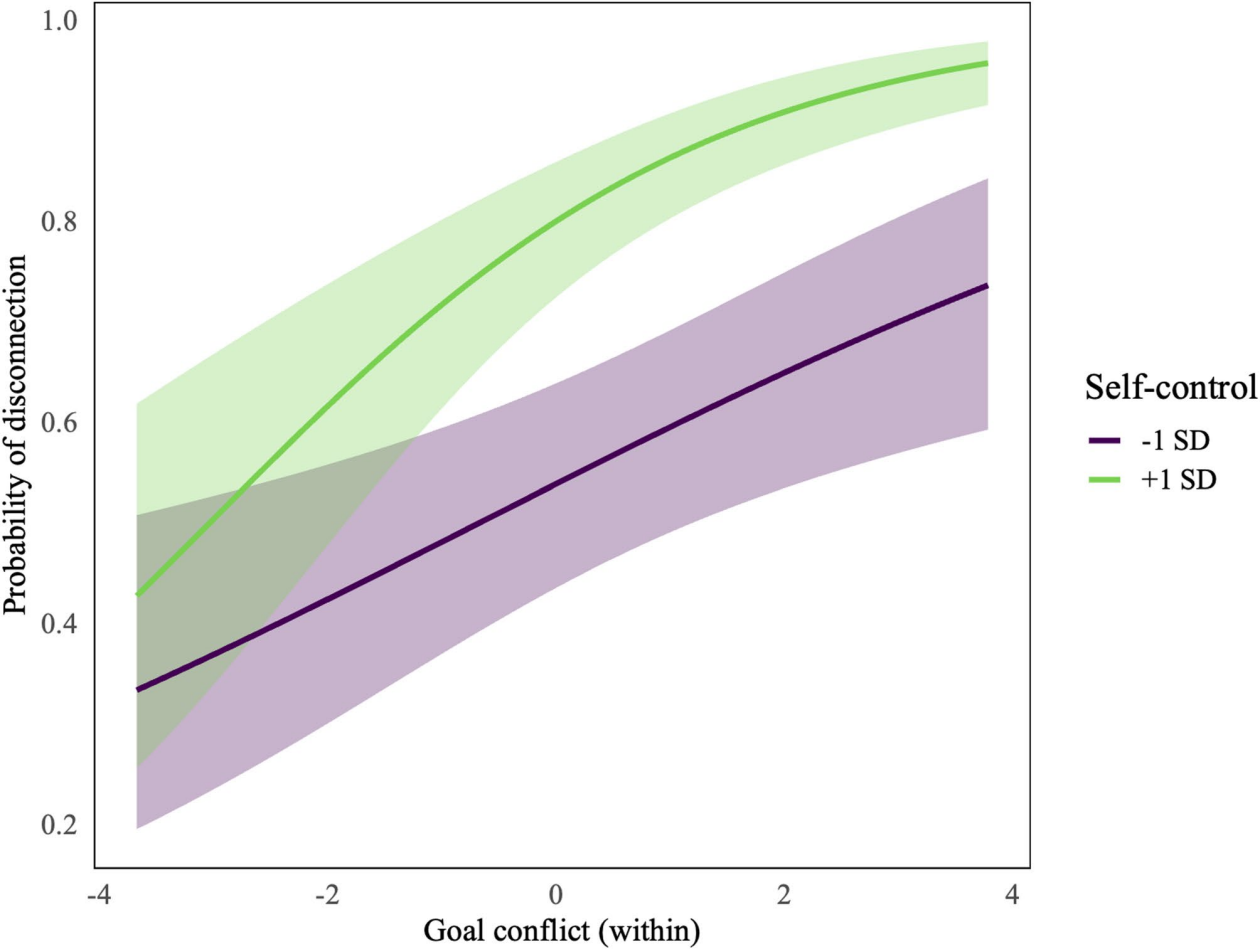
*Predicted probabilities of digital disconnection by the interaction of trait self-control and situational goal conflict.*

Figure 2 further indicates a significant interaction of trait self-control with situational goal conflict in the relationship with disconnection, supporting the hypothesized interaction effect, *b* = 0.11, OR = 1.12, 95% CI [1.00–1.25], *p* = .047. A simple slopes analysis depicted in Fig. 3 shows that for those higher in self-control (+ 1 SD), higher goal conflict is associated with an increased probability of disconnecting than for those lower in self-control (-1 SD).

Again, we constructed an exploratory multilevel model that mirrors the model depicted in Fig. 2, in this case predicting digital disconnection by goal conflict in the previous situation (*t*-1). The lag model indicates that high goal conflict in the previous situation makes disconnection 7% more likely in the following situation, i.e., at least two hours later, *b* = 0.07, OR = 1.07, 95% CI [1.01–1.13], *p* = .016. However, the interaction between goal conflict in the previous situation and trait self-control did not significantly predict disconnection in the lag model, *b* = -0.01, OR = 0.99, 95% CI [0.94–1.05], *p* = .758.

### Disconnection as a mechanism to reduce procrastination

**Fig. 4:**
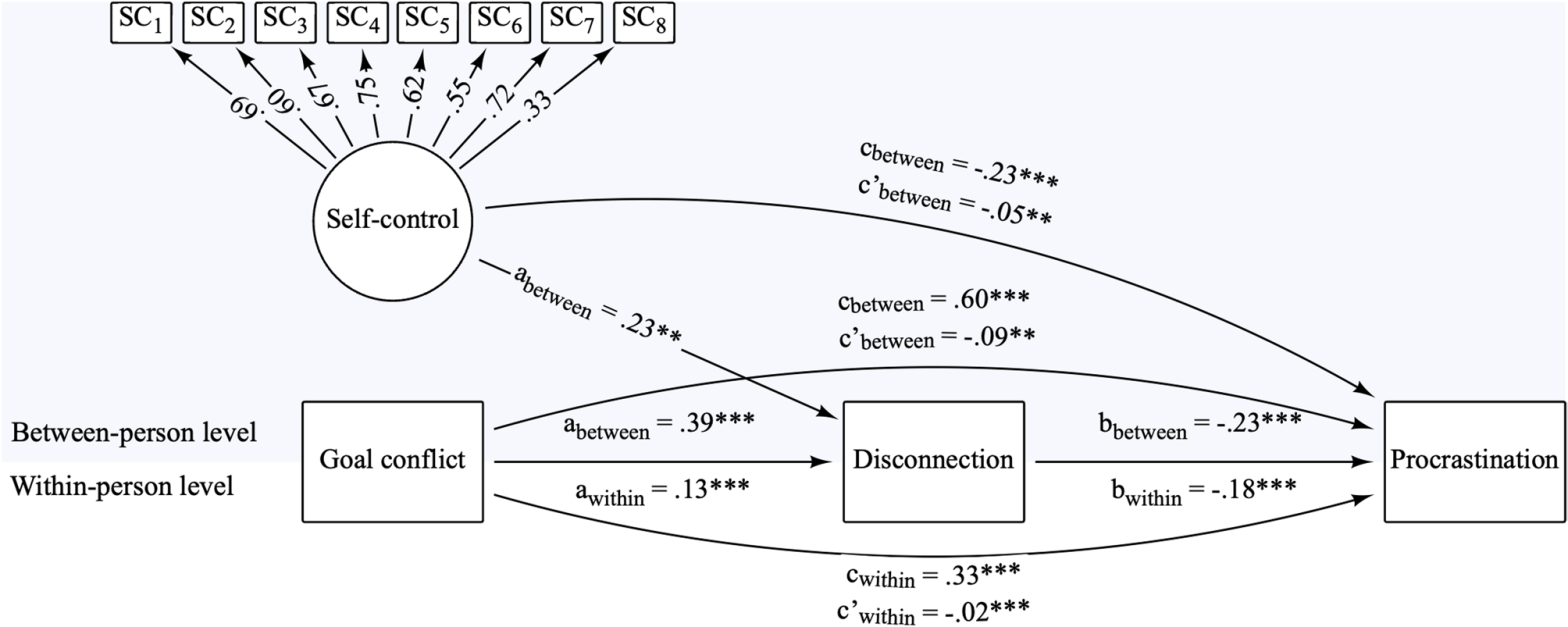
*Multilevel mediation structural equation model.*

*Notes*. Variables and coefficients in the area highlighted in light blue are on the between-person level (L2), variables and coefficients in the white area represent the within-person level (L1). Disconnection describes the breadth of disconnection behaviors. Variables measured at L1 were aggregated to a L2 measure by grand-mean centering. *N* = 236, *T* = 12,362. Model fit: CFI = 0.986, RMSEA = 0.009, SRMR = 0.075.

Next, we explored whether digital disconnection mediates the relationships between goal conflict and trait self-control with procrastination, again separating between- from within-person variance components. Accordingly, we specified a multilevel structural equation mediation model. As pre-registered, we use the mean score across all disconnection levels as the measure of disconnection to reflect the breadth of digital disconnection strategies that users engage in. A model without interactions shows acceptable fit, CFI = 0.986, RMSEA = 0.009, SRMR = 0.075. The path model depicted in Fig. 4 shows that goal conflict has a positive total effect on procrastination both at the between- and within-person level, indicating that individuals who experience more goal conflict than others procrastinate considerably more (*b* = 0.36, β = 0.60, *p* < .001) and that in situations in which goal conflict is more prevalent, procrastination with digital media tends to be moderately higher (*b* = 0.33, β = 0.33, *p* < .001). However, when including digital disconnection as a mediator, the indirect effect between goal conflict and procrastination is weakly negative at the between- (*b* = -0.05, β = − 0.09, *p* = .001) and within-person level (*b* = -0.02, β = − 0.02, *p* < .001). Breaking this mediation effect down to its individual steps, goal conflict is moderately positively associated with more disconnection behaviors at the between- (*b* = 0.07, β = 0.39, *p* < .001) and weakly at the within-person level (*b* = 0.02, β = 0.13, *p* < .001). In turn, the association of disconnection and procrastination is moderately negative both at the between- (*b* = -0.74, β = − 0.23, *p* < .001) and the within-person level (*b* = -0.93, β = − 0.18, *p* < .001). Overall, this pattern of findings indicates that engaging in more digital disconnection suppresses the detrimental associations between goal conflict and procrastination, lending further support for disconnection as an effective strategy to resolve goal conflicts and potentially reduce procrastination.

Additionally, the model finds that higher trait self-control is associated with lower procrastination, with a small to moderate negative total effect, *b* = − 0.22, β = − 0.23, *p* < .001. The indirect effect of trait self-control on procrastination via digital disconnection on the within-person level is smaller but still negative (*b* = -0.05, β = − 0.05, *p* = .010). Trait self-control also predicts slightly to moderately higher disconnection at the between-person level, *b* = 0.07, β = 0.23, *p* = .001. Because the factor loadings for two of the trait self-control items were below 0.60, we conducted a robustness check that excluded these items. The results were highly similar, and no conclusions changed between the two models. Results are available in the Open Science Framework (OSF):https://osf.io/m8pu3.

While the findings are correlational, they could indicate that temporary disconnection might be an effective mechanism through which those high in self-control reduce procrastination in an environment of constant connectivity.

### Levels of disconnection

Finally, to explore which forms of disconnection (e.g., device, app, or feature level) are associated with less procrastination, we computed a multilevel model that predicts procrastination from the within- and between-person components of each level of disconnection (binary predictors). Within participants, we find a small association between digital disconnection from devices and lower procrastination in the same situation, *b* = − 0.28, β = ‑0.06, 95% CI [‑0.08 – ‑0.05], *p* < .001. Similarly, in situations in which participants disconnect more from applications (*b* = − 0.13, β = − 0.03, 95% CI [‑0.05 – ‑0.01], *p* = .002) or specific features (*b* = − 0.20, β = ‑0.04, 95% CI [-0.06 – − 0.02], *p* < .001), they procrastinate less. Within participants, disconnection from interactions (*b* = 0.02, β = 0.00, 95% CI [-0.02 – 0.03], *p* = .668) or specific message content (*b* = − 0.09, β = − 0.02, 95% CI [‑0.04 – 0.00], *p* = .078) was not significantly associated with procrastination. At the between-person level, only disconnecting from features was related to moderately less procrastination (*b* = -1.60, β = − 0.29, 95% CI [-0.45 – − 0.14], *p* < .001), whereas no other disconnection level was significantly associated with procrastination between participants (βs ≤ 0.12, *ps* ≥ 0.060).

## Discussion

In the pervasive “attention economy”, individuals’ capacity to navigate ubiquitous digital media options is constantly challenged. This study investigated whether digital disconnection—voluntarily and temporarily reducing digital media use—could be employed as a self-control strategy to avoid procrastination. Our research takes a psychological, individual-level perspective on users’ self-regulation of their digital technology engagement^18^. Employing one of the first quantitative, in situ investigations into digital disconnection as an anti-procrastination strategy, our results indicate that disconnection was indeed associated with less procrastination in the same and the following situation a few hours later. Furthermore, we find that individuals were more likely to disconnect when trait self-control was higher, in situations with more goal conflict, and the more goal conflict they experienced on average in their daily lives. Our exploratory multilevel mediation analysis suggests that engaging in more digital disconnection could be a mechanism that helps resolve goal conflicts adaptively.

With these findings our study sheds light on a crucial, yet understudied strategy individuals in highly digitalized societies increasingly use to mitigate unwanted outcomes of digital media in their daily lives. Successful self-regulation is an important antecedent of well-being, achievement, and other crucial life outcomes. Thus, societal measures that improve the access to, ability for, and the right to digital disconnection may make an important contribution to well-being, productivity, work-life balance, and quality of life^60^.

By assessing digital disconnection and procrastination in-situ, this study helps us understand how self-regulatory mechanisms of digital technology use operate in daily life. Conducting an ESM study, we follow calls to adopt a multilevel perspective on self-regulation^61^ and disconnection^17^. Specifically, we distinguish digital disconnection behaviors at the between- and within-person level and how they may be predicted by trait self-control. Our results show that this nuanced situational perspective on digital disconnection and procrastination is warranted because (a) associations on the between-person level differ from within-person associations and (b) the majority of variance, both for digital disconnection behaviors and procrastination occurred at the within-person level (ICC = 0.41 and ICC = 0.25, respectively). This could indicate that digital disconnection is not just a result of high trait self-control^62^ but can also be viewed as a situational self-control strategy. Additional analyses show that digital disconnection was even associated with lower procrastination in the next situation, at least two hours later. This has two central implications. First, it suggests that our proposed temporal order—that disconnection precedes less procrastination—is supported. Second, it indicates that the benefits of digital disconnection persist for a while. For example, in a work context this would mean that disconnection benefits persisted for (at least) a quarter of an eight-hour workday.

An additional mediation analysis within a multilevel structural equation model serves as a further investigation into the mechanisms through which self-regulation of digital media use operates. Model findings support the assumption that goal conflicts between important tasks and more pleasurable and immediately rewarding media use makes procrastination more likely. However, when we include digital disconnection as a mediator, stronger goal conflicts are associated with more digital disconnection behaviors and disconnection is in turn associated with less procrastination. This pattern of findings overall indicates that using digital disconnection strategies may suppress negative consequences of goal conflicts.

Further, the exploration of different levels of digital disconnection shows that disconnecting was associated with slightly less procrastination at the device, application, and feature levels but not at the interaction or message content levels. This is in line with recent theoretical work suggesting that disconnection to reduce procrastination mainly operates on the device and application levels^17,63^. Although future confirmatory work is needed, we find initial support for this proposition. Beyond the device and application levels, we also find that disconnecting from certain features was associated with less procrastination. This could indicate that technological interventions, e.g. allowing users to set time limits on applications, can also be effective against procrastination.

Additionally, while channel-focused disconnection behaviors (i.e., targeting the device, application, and feature levels^34^ were associated with slightly less procrastination and communication-focused disconnection behaviors (i.e., targeting the interaction and message level^34^ were not, disconnecting from entire channels likely also targets communication-focused elements because they are nested within channels. Put simply, if one tends to procrastinate by watching short videos about cats, this might be more effectively prevented by avoiding opening *any* short-video app or by putting the phone away, rather than just trying to avoid cat videos. With smartphones and digital media being ‘meta-media’ that contain multitudes of potential interactions and communication content^64^ and the strongly habit-driven nature of procrastination with digital media^14^, preventing access to temptations in the first place seems to be an effective way to reduce procrastination. Research on self-control, more generally, suggests that effective self-control is particularly achieved by arranging situations preventively in such a way that temptations and goal conflict are less likely to arise^45,65–67^—rather than trying to intervene once a temptation has arisen. Therefore, while directly targeting disruptive interactions or content through digital disconnection could be a potentially viable strategy to reduce procrastination, preventing temptation through situation selection and modification at a higher level seems to be the more effective digital disconnection strategy^23,32^.

### Limitations and future directions

We assessed disconnection across all main levels and types of digital media use and how it is associated with procrastination. Future studies could investigate how effective specific strategies are at reducing procrastination, for example, strategies that address a certain aspect of media use (e.g., a specific app or feature) or strategies that reduce the attractiveness of alternatives to primary tasks^68^.

Based on prior theorizing, we expected a combination of high capacity (i.e. self-control) and relevant situational conditions (i.e. goal conflict) to lead to more efforts at self-regulation^24^. While we did find that trait self-control predicted more disconnection behaviors at the within-person level, the cross-level interaction between trait self-control and goal conflict was relatively small and only slightly below our threshold of practical significance. One explanation for the small effect size could be that if trait self-control is high, individuals are already very likely to employ (preventive) disconnection strategies, introducing potential ceiling effects^62^. To be more confident in this finding, sampling more observations or participants would allow us to better understand the complexity of interactions between the capacity for self-control and goal conflicts as well as other potentially relevant situational factors such as motivation or social contexts.

We measured digital disconnection and procrastination based on self-reports. In our conceptualizations, both digital disconnection and procrastination include subjective experiences, that is, digital disconnection is a *deliberate* reduction of digital media use and procrastination is a form of delay of intended tasks that is *perceived* as irrational. While for both disconnection and procrastination, subjective perceptions are the defining yardsticks, future studies could benefit from complementing them with *observed* disconnection and procrastination behaviors. For example, studies could investigate reductions in digital device usage through digital trace data or monitor actual work on a task. Additionally, future studies could include downstream outcomes of procrastination such as work performance, academic achievement, or well-being^26,69^.To reduce intrusiveness, we asked for participants’ retrospective of the last two hours. Thus, there can be delays between the situations we intend to assess and the actual measurement. Future studies could implement event-based assessment of digital disconnection to reduce recall bias.

Furthermore, this study uses participants’ phones to collect survey responses. Our goal was to reduce intrusiveness of the study by avoiding additional research devices or paper-pencil measurements. Further, we focused on *everyday* disconnection instead of longer but often artificial “digital detox” periods. Still, a potential drawback of our approach is that measuring digital disconnection via surveys on participants’ digital devices could affect their disconnection behavior^70^. While reactivity in ESM on digital media use tends to be relatively low^71^, future studies could employ alternative or complementary ways of data collection such as (paper-pencil) diaries, experiments, or combining self-reports with digital trace data.

In this work, we take a self-regulatory perspective on digital disconnection^18^. Such perspectives have been criticized for focusing on self-optimization and putting the responsibility for succeeding in the “attention economy” unfairly on individuals^72^. We agree. However, we argue that as problems with constant connectivity are prevalent, supporting users’ agency and finding ways for effective self-regulation is important. Still, future research should also consider broader societal or organizational solutions on a more systemic level that reduce technology demands and increase agency in individuals’ self-regulation, such as policies that regulate problematic features, the business activities of technology companies, or implementing worker protections such as a “right to disconnect”. Additionally, not all forms of task delay are problematic^73^. The present study focused on detrimental forms of task delay, with participants self-reporting whether they *needlessly* delayed tasks they intended to get done. Yet, the societal context, such as a dominant “culture of productivity”, may affect participants’ perceptions of what constitutes “good” and “rational” vs. “bad” and “irrational” task delay^74^. Norms around procrastination with digital media in highly digitalized societies seem particularly focused on productivity and likely play an important role in shaping goal hierarchies. Thus, future research should also critically investigate how such expectations affect perceptions of instrumental rationality in digital self-regulation^3,29^. Future studies could also take a functional perspective on media breaks, where task delay makes time for necessary recreational activities—including media use^75^—and how such recovery experiences relate to digital disconnection.

This study’s goal was to investigate the effectiveness of real-life disconnection behaviors through experience sampling. While this approach contributes important in situ evidence and we included key controls, it comes with limited internal validity because reverse causality and situational confounders can still be at play. Whereas the separation of between- and within-person variance allows for controlling between-person variance for within-person associations, between-person coefficients may be biased by between-person confounders. Additionally, uncontrolled situational factors (i.e., time-varying confounds) could bias within-person coefficients. Thus, future studies could benefit from additionally incorporating experimental elements, for example, micro-randomized trials that combine ESM with momentary experimental interventions^76^. These are important next steps to investigate causal mechanisms of self-regulation and establish the effectiveness of digital disconnection strategies against procrastination.

## Conclusion

In a world of highly digitalized everyday lives and prevalent goal conflicts, digital disconnection may offer an important pathway for effective self-regulation. We argue that digital disconnection could decrease procrastination, by helping media users resolve goal conflicts between situational desires and long-term goals. Our findings indeed suggest that reductions in digital media use may not have to be implemented as rigorous day- or week-long digital detox programs but can be effectively employed as an intentional situational strategy to reduce procrastination.

## Data Availability

Data and materials are available in the OSF at (https:/osf.io/m8pu3).

## References

[1] Wolf, R. D. & Vanden Abeele, M. M. P. Mobile media and social space: How anytime, anyplace connectivity structures everyday life. *Media Communication*. **6**, 5–14 (2018).

[2] Reinecke, L., Gilbert, A. & Eden, A. Self-regulation as a key boundary condition in the relationship between social media use and well-being. *Curr. Opin. Psychol.* **45**, 101296 (2022). 35085935 10.1016/j.copsyc.2021.12.008

[3] Van Bruyssel, S. & De Wolf, R. Vanden Abeele, M. Who cares about digital disconnection? Exploring commodified digital disconnection discourse through a relational lens. *Convergence: Int. J. Res. into New. Media Technol.* **13548565231206504**10.1177/13548565231206504 (2023).

[4] Vanden Abeele, M. M. P., Halfmann, A. & Lee, E. W. J. Drug, demon, or donut? Theorizing the relationship between social media use, digital well-being and digital disconnection. *Curr. Opin. Psychol.* **45**, 101295 (2022). 35123383 10.1016/j.copsyc.2021.12.007

[5] Klingsieck, K. B. Procrastination in different life-domains: Is procrastination domain specific? *Curr. Psychol.* **32**, 175–185 (2013).

[6] Inzlicht, M., Werner, K. M., Briskin, J. L. & Roberts, B. W. Integrating models of self-regulation. *Annu. Rev. Psychol.* **72**, 319–345 (2021). 33017559 10.1146/annurev-psych-061020-105721

[7] Steel, P. The nature of procrastination: A meta-analytic and theoretical review of quintessential self-regulatory failure. *Psychol. Bull.* **133**, 65–94 (2007). 17201571 10.1037/0033-2909.133.1.65

[8] Aalbers, G., Vanden Abeele, M. M. P., Hendrickson, A. T., Marez, L. & Keijsers, L. Caught in the moment: Are there person-specific associations between momentary procrastination and passively measured smartphone use? *Mob. Media Communication*. **10**, 115–135 (2022).

[9] Meier, A., Reinecke, L. & Meltzer, C. E. Facebocrastination? Predictors of using Facebook for procrastination and its effects on students’ well-being. *Comput. Hum. Behav.* **64**, 65–76 (2016).

[10] Bayer, J. B., Anderson, I. A. & Tokunaga, R. S. Building and breaking social media habits. *Curr. Opin. Psychol.* **45**, 101303 (2022). 35255413 10.1016/j.copsyc.2022.101303

[11] Flayelle, M. et al. A taxonomy of technology design features that promote potentially addictive online behaviours. *Nat. Rev. Psychol.* **2**, 136–150 (2023).

[12] Meier, A., Beyens, I., Siebers, T., Pouwels, J. L. & Valkenburg, P. M. Habitual social media and smartphone use are linked to task delay for some, but not all, adolescents. *J. Computer-Mediated Communication*. **28**, zmad008 (2023).

[13] Meier, A. Studying problems, not problematic usage: Do mobile checking habits increase procrastination and decrease well-being? *Mob. Media Communication*. **10**, 272–293 (2022).

[14] Schnauber-Stockmann, A., Meier, A. & Reinecke, L. Procrastination out of habit? The role of impulsive versus reflective media selection in procrastinatory media use. *Media Psychol.* **21**, 640–668 (2018).

[15] Klingelhoefer, J., Gilbert, A. & Meier, A. Momentary motivations for digital disconnection: an experience sampling study. *J. Computer-Mediated Communication*. **29**, zmae013 (2024).

[16] Nassen, L. M., Vandebosch, H., Poels, K. & Karsay, K. Opt-out, abstain, unplug. A systematic review of the voluntary digital disconnection literature. *Telematics Inform.* **81**, 101980 (2023).

[17] Vanden Abeele, M. M. P. et al. Why, how, when, and for whom does digital disconnection work? A process-based framework of digital disconnection. *Communication Theory* qtad016 (2024). 10.1093/ct/qtad016

[18] Ross, M. Q. et al. Mapping a pluralistic continuum of approaches to digital disconnection. *Media Cult. Soc.* **46**, 851–862 (2024).

[19] Hinsch, C. & Sheldon, K. M. The impact of frequent social Internet consumption: Increased procrastination and lower life satisfaction. *J. Consumer Behav.* **12**, 496–505 (2013).

[20] Lemahieu, L. et al. The effects of social media abstinence on affective well-being and life satisfaction: a systematic review and meta-analysis. *Sci. Rep.* **15**, 7581 (2025). 40038410 10.1038/s41598-025-90984-3 PMC11880199

[21] Vanden Abeele, M. M. P., Murphy, S. L., Lemahieu, L. & Koster, E. H. W. Methodological considerations for social media intervention studies. *Nat. Rev. Psychol.* **4**, 603–614 (2025).

[22] Bolger, N., Davis, A. & Rafaeli, E. Diary Methods: Capturing Life as it is Lived. *Annu. Rev. Psychol.* **54**, 579–616 (2003). 12499517 10.1146/annurev.psych.54.101601.145030

[23] Parry, D. A., Kuit, C., Murray, A. & van der Westhuizen, A. Connect to disconnect: What an online community for digital disconnection can tell us about digital well-being. *New. Media Soc.* **14614448251362436**10.1177/14614448251362436 (2025).

[24] Kotabe, H. P. & Hofmann, W. On Integrating the Components of Self-Control. *Perspect. Psychol. Sci.* **10**, 618–638 (2015). 26386000 10.1177/1745691615593382

[25] Steel, P., Svartdal, F., Thundiyil, T. & Brothen, T. Examining procrastination across multiple goal stages: A longitudinal study of temporal motivation theory. *Front Psychol* **9**, (2018). 10.3389/fpsyg.2018.00327 PMC5891720 29666590

[26] Sirois, F. M. & Pychyl, T. Procrastination and the priority of short-term mood regulation: Consequences for future self. *Soc. Pers. Psychol. Compass*. **7**, 115–127 (2013).

[27] Stanovich, K. E., Toplak, M. E. & West, R. F. The Development of Rational Thought: A Taxonomy of Heuristics and Biases. in *Advances in Child Development and Behavior* vol. 36 251–285JAI, (2008). 10.1016/s0065-2407(08)00006-2 18808045

[28] Kühnel, J., Bledow, R. & Kuonath, A. Overcoming procrastination: Time pressure and positive affect as compensatory routes to action. *J. Bus. Psychol.* **38**, 803–819 (2023).

[29] Giguère, B., Sirois, F. M. & Vaswani, M. Delaying Things and Feeling Bad About It? A Norm-Based Approach to Procrastination. in *Procrastination, Health, and Well-Being* 189–212 (Elsevier, 2016). 10.1016/B978-0-12-802862-9.00009-8

[30] Wiley, A. The Grind Never Stops Mental Health and Expectations of Productivity in the North American University. *anthropologica* 65, (2023).

[31] Corkin, D. M., Yu, S. L. & Lindt, S. F. Comparing active delay and procrastination from a self-regulated learning perspective. *Learn. Individual Differences*. **21**, 602–606 (2011).

[32] Duckworth, A. L., Gendler, T. S. & Gross, J. J. Situational strategies for self-control. *Perspect. Psychol. Sci.* **11**, 35–55 (2016). 26817725 10.1177/1745691615623247 PMC4736542

[33] Gilbert, A., Reinecke, L., Meier, A., Baumgartner, S. E. & Dietrich, F. Too amused to stop? Self-control and the disengagement process on Netflix. *J. Communication*. **74**, 387–398 (2024).

[34] Meier, A. & Reinecke, L. Computer-mediated communication, social media, and mental health: A conceptual and empirical meta-review. *Communication Res.* **48**, 1182–1209 (2021).

[35] Radtke, T., Apel, T., Schenkel, K., Keller, J. & Von Lindern, E. Digital detox: An effective solution in the smartphone era? A systematic literature review. *Mob. Media Communication*. **10**, 190–215 (2022).

[36] Klingelhoefer, J., Nguyen, M. H., Dr. & Geber, S. Do Disconnection Strategies Improve Well-Being and Productivity? A Two-Wave Panel Study Over One Month. Preprint at https://osf.io/preprints/psyarxiv/27n3w_v1/ (2026).

[37] Reinecke, L. & Hofmann, W. Slacking off or winding down? An experience sampling study on the drivers and consequences of media use for recovery versus procrastination. *Hum. Commun. Res.* **42**, 441–461 (2016).

[38] Cobb-Clark, D. A., Dahmann, S. C., Kamhöfer, D. A. & Schildberg-Hörisch, H. The predictive power of self-control for life outcomes. *J. Econ. Behav. Organ.* **197**, 725–744 (2022).

[39] Nielsen, K. S., Gwozdz, W. & De Ridder, D. Unraveling the relationship between trait self-control and subjective well-being: The mediating role of four self-control strategies. *Front Psychol* **10**, (2019). 10.3389/fpsyg.2019.00706 PMC6445882 30971998

[40] Nguyen, M. H., Büchi, M. & Geber, S. Everyday disconnection experiences: Exploring people’s understanding of digital well-being and management of digital media use. *New. Media Soc.* **26**, 3657–3678 (2024).

[41] Halfmann, A., Meier, A. & Reinecke, L. Too much or too little messaging? Situational determinants of guilt about mobile messaging. *J. Computer-Mediated Communication*. **26**, 72–90 (2021).

[42] Halfmann, A., Meier, A. & Reinecke, L. Trapped Between Goal Conflict and Availability Norm? *J. Media Psychol.* **36**, 45–57 (2024).

[43] Orhan, M. A., Castellano, S., Khelladi, I., Marinelli, L. & Monge, F. Technology distraction at work. Impacts on self-regulation and work engagement. *J. Bus. Res.* **126**, 341–349 (2021).

[44] Wenzel, M., Bürgler, S., Brandstätter, V., Kreibich, A. & Hennecke, M. Self-regulatory strategy use, efficacy, and strategy-situation-fit in self-control conflicts of initiation, persistence, and inhibition. *Eur. J. Pers.* **38**, 189–208 (2024). 41908315 10.1177/08902070221150478 PMC13021067

[45] Hofmann, W., Baumeister, R. F., Förster, G. & Vohs, K. D. Everyday temptations: an experience sampling study of desire, conflict, and self-control. *J. Personal. Soc. Psychol.* **102**, 1318–1335 (2012). 10.1037/a0026545 22149456

[46] Haynes, A., Kemps, E., Moffitt, R. & Mohr, P. Resisting temptation of unhealthy food: interaction between temptation-elicited goal activation and self-control. *Motiv Emot.* **38**, 485–495 (2014).

[47] Hofmann, W., Gschwendner, T., Friese, M., Wiers, R. W. & Schmitt, M. Working memory capacity and self-regulatory behavior: Toward an individual differences perspective on behavior determination by automatic versus controlled processes. *J. Personal. Soc. Psychol.* **95**, 962–977 (2008). 10.1037/a0012705 18808271

[48] Soravia, L. M., Schläfli, K., Stutz, S., Rösner, S. & Moggi, F. Resistance to temptation: The interaction of external and internal control on alcohol use during residential treatment for alcohol use disorder. *Alcoholism Clin. Exp. Res.* **39**, 2209–2214 (2015). 10.1111/acer.12880 PMC5057333 26503067

[49] J Leiner, D. Our research’s breadth lives on convenience samples A case study of the online respondent pool SoSci Panel. *SCM* **5**, 367–396 (2016).

[50] FAQ. Humanities and Social Sciences. https://www.dfg.de/en/research-funding/proposal-funding-process/faq/humanities-social-sciences

[51] SoSci Panel. SoSci Panel für Wissenschaftlerinnen und Wissenschaftlern. *SoSci Panel - offenes wissenschaftliches Befragungspanel* https://www.soscipanel.de/researchers.php

[52] Klein, E. M. et al. Assessing procrastination: Dimensionality and measurement invariance of the general procrastination scale – screening (GPS-S) in a representative sample. *Eur. J. Psychol. Assess.* **35**, 633–640 (2019).

[53] Sirois, F. M., Yang, S. & van Eerde, W. Development and validation of the General Procrastination Scale (GPS-9): A short and reliable measure of trait procrastination. *Pers. Indiv. Differ.* **146**, 26–33 (2019).

[54] Gilbert, A., Baumgartner, S. E. & Reinecke, L. Situational boundary conditions of digital stress: Goal conflict and autonomy frustration make smartphone use more stressful. *Mob. Media Communication*. **11**, 435–458 (2023).

[55] Bertrams, A. & Dickhäuser, O. Messung dispositioneller Selbstkontroll-Kapazität. *Diagnostica* **55**, 2–10 (2009).

[56] Maloney, P. W., Grawitch, M. J. & Barber, L. K. The multi-factor structure of the Brief Self-Control Scale: Discriminant validity of restraint and impulsivity. *J. Res. Pers.* **46**, 111–115 (2012).

[57] Bates, D., Mächler, M., Bolker, B. & Walker, S. Fitting linear mixed-effects models using lme4. *J Stat. Soft* **67**, (2015).

[58] Bell, A., Fairbrother, M. & Jones, K. Fixed and random effects models: Making an informed choice. *Qual. Quant.* **53**, 1051–1074 (2019).

[59] Rosseel, Y. & lavaan An R package for structural equation modeling. *J Stat. Soft* **48**, (2012).

[60] European Foundation for the Improvement of Living and Working Conditions. *Right to Disconnect: Implementation and Impact at Company Level* (Publications Office, 2024).

[61] Hofmann, W. Going beyond the individual level in self-control research. *Nat. Rev. Psychol.* **3**, 56–66 (2024).

[62] Inzlicht, M. & Roberts, B. W. The fable of state self-control. *Curr. Opin. Psychol.* **58**, 101848 (2024). 39096668 10.1016/j.copsyc.2024.101848

[63] Gilbert, A., Klingelhoefer, J. & Meier, A. Disconnect to recharge: Well-being benefits of digital disconnection in daily life. *Communication Res.*10.1177/00936502251387830 (2025).

[64] Humphreys, L., Karnowski, V. & von Pape, T. Smartphones as metamedia: A framework for identifying the niches structuring smartphone use. *Int. J. Communication*. **12**, 2793–2809 (2018).

[65] Ent, M. R., Baumeister, R. F. & Tice, D. M. Trait self-control and the avoidance of temptation. *Pers. Indiv. Differ.* **74**, 12–15 (2015).

[66] Gillebaart, M. & De Ridder, D. T. D. Effortless self-control: A novel perspective on response conflict strategies in trait self‐control. *Social Personality Psych*. **9**, 88–99 (2015).

[67] Hofmann, W., Luhmann, M., Fisher, R. R., Vohs, K. D. & Baumeister, R. F. Yes, but are they happy? Effects of trait self-control on affective well-being and life satisfaction. *J. Pers.* **82**, 265–277 (2014). 23750741 10.1111/jopy.12050

[68] Dekker, C. A. & Baumgartner, S. E. Is life brighter when your phone is not? The efficacy of a grayscale smartphone intervention addressing digital well-being. *Mob. Media Communication*. **12**, 688–708 (2024).

[69] Herzog-Krzywoszanska, R., Krzywoszanski, L. & Kargul, B. General procrastination and bedtime procrastination as serial mediators of the relationship between temporal perspective and sleep outcomes. *Sci. Rep.* **14**, 31175 (2024). 39732750 10.1038/s41598-024-82523-3 PMC11682291

[70] Nguyen, M. H. & Hargittai, E. Digital disconnection, digital inequality, and subjective well-being: A mobile experience sampling study. *J. Computer-Mediated Communication*. **29**, zmad044 (2024).

[71] Toth, R., Parry, D. A. & Klingelhoefer, J. Somebody’s (still) watching me: Reactivity to smartphone logging and experience sampling. *Preprint at.*10.31235/osf.io/xt24p_v3 (2025).

[72] Enli, G. & Fast, K. Political Solutions or user Responsibilization? How Politicians understand Problems Connected to Digital Overload. *Convergence: Int. J. Res. into New. Media Technol.* **29**, 675–689 (2023).

[73] Cao, L. Examining ‘active’ procrastination from a self-regulated learning perspective. *Educational Psychol.* **32**, 515–545 (2012).

[74] Bozan, V. & Treré, E. The Politics of Disconnective Media: Unraveling the Materiality of Discourses on Disconnectivity. *MaC* **12**, 8586 (2024).

[75] Rieger, D., Hefner, D. & Vorderer, P. Mobile recovery? The impact of smartphone use on recovery experiences in waiting situations. *Mob. Media Communication*. **5**, 161–177 (2017).

[76] Marciano, L., Jindal, S. & Viswanath, K. Digital detox and well-being. *Pediatrics* **154**, e2024066142 (2024). 39285845 10.1542/peds.2024-066142 PMC11422191

